# Blockade of beta adrenergic receptors protects the blood brain barrier and reduces systemic pathology caused by HIV-1 Nef protein

**DOI:** 10.1371/journal.pone.0259446

**Published:** 2021-11-16

**Authors:** Jocelyn Rivera-Ortiz, Jessalyn Pla-Tenorio, Myrella L. Cruz, Krystal Colon, Jaileene Perez-Morales, Julio A. Rodriguez, Jorge Martinez-Sicari, Richard J. Noel

**Affiliations:** 1 HIV-1 Immunopathogenesis Laboratory, The Wistar Institute, Philadelphia, PA, United States of America; 2 Department of Basic Sciences, Ponce Health Sciences University, Ponce Research Institute, Ponce, Puerto Rico; 3 Cooper University Hospital Department of Orthopaedic Surgery, Camden, NJ, United States of America; Lewis Katz School of Medicine, Temple University, UNITED STATES

## Abstract

Combination antiretroviral therapy (cART) targets viral replication, but early viral protein production by astrocytes may still occur and contribute to the progression of HIV-1 associated neurocognitive disorders and secondary complications seen in patients receiving cART. In prior work with our model, astrocytic HIV-1 Nef expression exhibits neurotoxic effects leading to neurological damage, learning impairment, and immune upregulation that induces inflammation in the lungs and small intestine (SI). In this follow-up study, we focus on the sympathetic nervous system (SNS) as the important branch for peripheral inflammation resulting from astrocytic Nef expression. Male and female Sprague Dawley rats were infused with transfected astrocytes to produce Nef. The rats were divided in four groups: Nef, Nef + propranolol, propranolol and naïve. The beta-adrenergic blocker, propranolol, was administered for 3 consecutive days, starting one day prior to surgery. Two days after the surgery, the rats were sacrificed, and then blood, brain, small intestine (SI), and lung tissues were collected. Levels of IL-1β were higher in both male and female rats, and treatment with propranolol restored IL-1β to basal levels. We observed that Nef expression decreased staining of the tight junction protein claudin-5 in brain tissue while animals co-treated with propranolol restored claudin-5 expression. Lungs and SI of rats in the Nef group showed histological signs of damage including larger Peyer’s Patches, increased tissue thickness, and infiltration of immune cells; these findings were abrogated by propranolol co-treatment. Results suggest that interruption of the beta adrenergic signaling reduces the peripheral organ inflammation caused after Nef expression in astrocytes of the brain.

## Introduction

With the development of combination antiretroviral therapy (cART), people living with human immunodeficiency virus (HIV) have increased life expectancy. Despite successfully controlling systemic viremia, patients often develop comorbid pathologies such as interstitial pneumonitis, gut mucosal barrier dysfunction, and autonomic neuropathy [1–3]. However, these HIV-associated pathologies may not necessarily result from the viral replication, given the observed low plasma virus levels. Instead, they may result from inflammatory cytokine levels that remain elevated due to viral proteins still present in the body. For example, HIV-positive men receiving cART have elevated TNFα, IL-8, CCL13, and IL-12p70 compared to HIV-negative men [4]. Also, infected women receiving cART present elevated levels of TNFα and IL-1β [5]. In a study involving macaques with SIV-induced encephalitis, rapid progression to Acquired Immune Deficiency Syndrome (AIDS) correlated with an increase in IL-1β, IL-2, IL-6, IL-10, and TNF-α [6]. Thus, HIV viral proteins are an essential topic to study due to their toxic effects, including inflammation.

The blood brain barrier (BBB) integrity is known to be reduced due to tight junction protein disruption by several HIV proteins, and thereby a compromised BBB may cause inflammatory cytokines to cross over more abundantly from the periphery to the brain [7–10]. Cytokines such as interleukin IL-1β or TNF-α are inflammatory communicators from the immune system to the central nervous system (CNS) [11]. Robust signals of inflammatory cytokines, such as IL-1β, can lead to subsequent release of norepinephrine (NE) and epinephrine (Epi), inducing activation of the sympathetic nervous system (SNS) [12,13]. The SNS innervates both primary and secondary lymphoid organs contributing to the maturation, differentiation, and modulation of immune cells. The SNS also regulates physiological homeostasis and the responses to stressors serving as the communicator between the brain and periphery to regulate adaptive and innate responses [14,15]. Catecholamines are the neurotransmitters that modulate the adrenergic system via adrenergic receptors. The adrenoreceptors are found on different cell surfaces such as B-cells, macrophages, and astrocytes and work through nine different subtypes [16–18]. In HIV patients that died from encephalitis, beta-2 adrenergic receptor gene expression was upregulated throughout the frontal lobe white matter in HIV-positive patients undergoing cART, suggesting an adrenergic modulation [19]. Further, catecholamines increase the viral load, and cause SNS and hypothalamic-pituitary-adrenal axis dysfunction in HIV patients [11,20].

Our lab is interested in inflammation and toxicities produced by early viral proteins expressed despite the suppression of viral replication. HIV-1 Nef is an early protein (27-35kDa) that interacts with the host cell membrane and cytoplasmic proteins to enhance virus production in vivo [21]. This protein’s primary function is to induce endocytosis of CD4 and MHC-I receptors, resulting in an inhibited immune response [22]. Studies suggest that Nef protein can be found in macrophages and exosomes in patients’ serum, affecting normal biological processes [23,24]. In human brain tissue, Nef is produced by astrocytes despite a relative inability for productive viral replication seen in T-cells and macrophages [25]. However, a recent comprehensive study indicates that infection of astrocytes is sufficient to support migration of virus to the periphery, meaning that astrocyte infection may be important to maintaining a functional HIV reservoir in the brain and in peripheral organs [26]. Astrocytes expressing Nef in the hippocampus of rats are sufficient to cause spatial and recognition memory impairment, an increase in CCL2 in the brain, and increased hippocampus infiltration by CD163+ macrophages [27]. At least some components in the pathophysiology of Nef are mediated by activating the immune system and disrupting the BBB [28–30]. However, most studies concentrate on the effect of Nef in the brain, with few studies examining how those effects extend to the periphery. Recently, we demonstrated that the astrocytic expression of Nef could lead to BBB disruption, claudin-5 reduction, elevated serum IL-1β, and upregulated expression of macrophages in the lung and ileum of rats exposed to the protein [10]. Therefore, the pathway by which centrally expressed Nef could be inducing systemic inflammation is an area of interest.

In the present study which is a follow up to our earlier work with this model [10,27], we aimed to characterize the role of Nef in the activation of the SNS response. We chose to study the beta-adrenergic receptors (β-ARs) since activation of the G protein-coupled stimulatory receptor (GPCR) mediates the release of several inflammatory cytokine through activation of the NF-κB pathway [31]. In our *in-vivo* model we infused astrocytes expressing the Nef protein in the hippocampal area, where adrenergic fibers and cells that express adrenergic receptors are found [32]. We hypothesized that blocking non-selective β-ARs would inhibit tight junction protein disruption caused by Nef expression in astrocytes and reduce inflammation of distinct vital organs in the periphery. To test the adrenergic response, we treated the rats with propranolol, a non-selective β-AR antagonist. Propranolol is proven to be the antagonist of the β1 and β2-ARs, and the most widely used pharmacological agent to understand the effect of catecholamines [33,34]. We demonstrated that antagonization of the β-ARs in the presence of astrocytic Nef expression results in restored basal levels of IL-1β in serum, and normal expression of the tight junction protein claudin-5, small intestine, and lung tissue.

## Methods

### Institutional Animal Care and Use Committee (IACUC)

All protocols involving animals were approved by the Ponce Health Sciences University (IACUC) following the Laboratory Animal Welfare Act protocol 226. The animals were housed in pairs under a constant environmental condition with a 12-hour light-dark cycle. Standard laboratory rat chow and water was unrestricted throughout the study. To minimize pain and discomfort during surgical procedures, we administered inhalation anesthesia and post-operative application of Neosporin and benzocaine. Animals were observed daily by staff, including veterinary supervision. The parameters to determine the humane endpoint of the study were a failure to groom, move, or feed.

### Primary rat astrocyte extraction and culture

Primary rat astrocytes were obtained from Sprague Dawley rat brains. Rats were anesthetized with pentobarbital and decapitated for brain removal. Brain tissue was minced and disrupted in ice-cold Hank’s balanced salt solution with trypsin (Sigma, St. Louis, MO). Debris was removed from cells by sedimentation, and cells were recovered from the suspended phase above the settled debris. The process was repeated, discarding the first three extractions to reduce the presence of blood vessels and red blood cells. Cells were pooled and seeded in flasks with growth medium (Dulbecco’s Modified Eagle Media (DMEM) (Sigma) supplemented with 10% fetal bovine serum, 10 mM L-glutamine, 5% non-essential amino acids, and streptomycin/penicillin) and incubated at 37°C and 5% CO_2_. The non-attached cells were removed after three days in culture. Cells were cultured and used in experiments once three passages were completed to assure the cultures were astrocytes (positive staining of all cells for GFAP; neither neurons nor microglia were detectable by immunoblotting for cell-specific markers MAP-2 and Iba-1, respectively).

### Transfection of primary rat astrocytes and hippocampal infusion of astrocytes

Primary astrocytes were transfected with plasmid encoding Nef (p96AM651 NIH AIDS Reference Research and Reagent Program, Cat. # 8677). After transfection, the cells were re-suspended in sterile artificial cerebrospinal fluid (ACSF 1X) at a final concentration of 100,000 cells per 0.5uL of ACSF. Transfected astrocytes were used for a single infusion into the right hemisphere. The surgery was performed using 30-day old male and female Sprague Dawley rats that were anesthetized by isoflurane inhalation. Anesthetized rats received microinfusion using the following coordinates: anterior-posterior = -0.28, medial-lateral = +/-0.17, and dorsal-ventral = -0.37. Once the coordinates were set in the stereotaxic apparatus, we infused 0.5ul of transfected astrocytes; the needle was left in place for 5 minutes to eliminate backflow of cells. Afterward, the skull was covered with bone wax, and the scalp incision was sutured. A triple antibiotic ointment with an analgesic was applied to the surgical site to reduce pain and to aid with the recovery from the surgery.

### Cell immunofluorescence and Western blot

Cells were fixed in 2% paraformaldehyde solution at the time of collection and stored at 4°C. Cells were permeabilized with 0.5% Triton in phosphate buffered saline (PBS) for 20 minutes and blocked with 2% BSA in PBS for 30 minutes. Moreover, cells were stained with (1:100) dilution of anti-HIV-1 SF2 Nef Monoclonal (EH1) primary antibody (NIH AIDS Reagent Program, Cat#:3689) and left for 1 hour. Secondary antibody, Goat anti-mouse IgG (H+L) highly cross absorbed, Alexa Fluor 594 (ThermoFisher Scientific, Waltham,MA) was left incubating for 1 hour. After washing, cells were counterstained with DAPI (ThermoFisher Scientific), and the slides were analyzed with EVOS FL Imagine System (ThermoFisher Scientific).

For protein analysis cells were lysed 48 hours post transfection with radioimmunoprecipitation assay (RIPA) buffer (150mM sodium chloride, 1.0% Nonidet 40, 0.5% sodium deoxycholate, 0.1% sodium dodecyl sulfate, 50mM Tris, pH 8) containing protease and phosphatase inhibitors for further protein expression analysis. Protein samples for Nef 48 hours and Nef + Propranolol 48 hours were adjusted to 25μg in 25 μL 1x Laemmli buffer. All samples were heated for 5 minutes at 95°C. Samples were run on Any kD Mini-Protean TGX precast polyacrylamide gel (Biorad, Hercules,CA) and then transferred to PVDF membranes using the Mini-Protean II Cell Electrophoresis chamber. Membranes were blocked with 5% BSA in TBST for 1 hour at room temperature, followed by incubation overnight at 4°C with (1:1000) dilution of anti-HIV-1 SF2 Nef Monoclonal (EH1) primary antibody (NIH AIDS Reagent Program, Cat#:3689) and 1:5000 dilution monoclonal Anti-β-Actin (Sigma) in 5% BSA in TBST. The next day, membranes were washed two times with TBST, two times with 5% BSA in TBST, and incubated for 1 hour at room temperature with (1:5000) dilution of Anti-mouse IgG (GE-Healthcare, Chicago, IL) secondary antibody in 5% BSA in TBST. Membranes were again washed two times with 5% BSA in TBST and two times with TBST. The Amersham ECL Western Blotting Detection Reagents (GE-Healthcare) was used following the manufacturer’s specifications. The X-ray film (GE-Healthcare) developing was performed in the following manner: 7 minutes exposure, 3 minutes in the developer, 1 minute in the fixer, and was finally washed with water.

### Propranolol administration

Sprague Dawley rats of approximately 30 days of age, using equal numbers of males and females were divided into four groups. The four groups were: Nef, Nef + propranolol, propranolol, and Naïve, see S1 Table for summary of rats used in each figure. We wanted to determine if the effect of Nef required the sympathetic nervous system (SNS), specifically the beta-adrenergic receptor. To test this concept, we pre-treated the animals with beta-blocker propranolol to understand the effect of Nef in SNS. Intraperitoneal injections of propranolol (Sigma Aldrich) 10mg/kg or saline (Fisher, Hampton, NH) were administered to the rats one day before, the day of surgery, and post-operative day one. Two days after surgery, rats were euthanized via intraperitoneal pentobarbital overdose (65 mg/kg i.p.).

### ELISA

Serum was isolated from blood samples that were collected via a right ventricular puncture during sacrifice. The blood was incubated at room temperature for 30 minutes after being collected to promote coagulation. The blood was centrifuged at 10,000 revolutions per minute (RPM) for 10 minutes to separate the serum. Serum was stored in aliquots at -20°C. Levels of the pro-inflammatory cytokine, IL-1β, in blood were quantified in serum by enzyme-linked immunosorbent assay (ELISA) (eBioscience San Diego, CA) following the protocol provided by the manufacturer. Serum Nef was measured by a Nef-capture ELISA (ImmunoDx, Woburn, MA). For both ELISAs: samples were run in duplicate, the absorbance was measured using a BioTek Synergy HT multimode plate reader, and concentrations were calculated via interpolation of obtained values on a standard curve.

### Hematoxylin and eosin staining

Small intestine and lung tissue were fixed in formalin and paraffin embedded. Standard hematoxylin and eosin (H&E, ThermoFisher, Waltham, MA) staining was performed, followed by an examination of microscopic changes. The size of the Peyers Patches (PP) in the small intestine is an indicator of immune activation. In the small intestine, we analyzed stained tissues via microscope to assess the PP diameter attempting to observe altered immune activation based on different treatments.

### Immunofluorescence of CD68, iNOS, and claudin-5

Brain, small intestine, and lung tissues were preserved in formalin, paraffin embedded, cut at 4 um and mounted on positively charged slides. Brain slices were stained with anti-claudin-5 antibody. The slides were deparaffinized with xylol for 30 minutes following rehydration with ethanol at 100%, 95%, 80% and 70% sequentially for 3 minutes each. Then, the slides were washed with distilled water for 1 minute, followed by PBS for 5 minutes. Antigen retrieval was accomplished in EDTA, pH = 6.5, for 40 minutes at 98°C. The slides were left for 20 minutes at room temperature. Slides were washed with distilled water for 4 minutes and PBS for 5 minutes. After 15 minutes, a protein block solution was added to the tissue and left overnight with primary antibody claudin-5, 1:100, (Invitrogen, Carlsbad, CA) in a humidified chamber at 4°C before the addition of secondary antibody, Alexa Fluor 488 Goat Anti-mouse (Invitrogen). Finally, slides were incubated with DAPI (1uL DAPI 1:50) for 5 minutes. Two rounds of PBS washes were performed before the addition of mounting media. The double immunofluorescence in the small intestine with CD68 and iNOS was performed following the protocol described by [35]. CD68^+^A and iNOS cells were quantified using three randomly selected high-power fields (HPFs, image taken at 40x magnification) per rat. Images were taken by an experimenter blinded to treatment groups. CD68 staining was performed in small intestine and lung tissues. CD68 and iNOS staining was performed only in small intestine. Tissue florescence analyses were completed using the cell-counter plug-in of the ImageJ software package (version 1.42, NIH, USA). The images were taken using an Olympus BX60 microscope and NIS-Elements software package (Nikon, Minato, Japan) for image overlaying. Immunofluorescence analysis was performed using the software Image J (NIH).

### Statistical analysis

The statistical analysis was performed with GraphPad PRISM version 7 (La Jolla, Ca) and Microsoft Excel. Error bars represent the means ± S.E.M. All p-values were two-tailed and statistical significance was defined as: *p-value <0.05, **p-values <0.01, *** p-values <0.001, and **** p-values <0.0001. Outlier analysis test was performed on IL-1β and CD68 analysis on GraphPad PRISM 7 using the ROUT method, which can identify any number of outliers using a Q of 1% for both males and females. When comparing two groups, a non-parametric Mann Whitney test was performed to compare data among independent groups. A non-parametric Kruskal Wallis test was used to compare data within related groups. We also performed linear regression analyses on Microsoft Excel Data Analysis tool pack to analyze the association among treatment, gender, and IL-1β, a backward elimination was performed, and factors are considered for removal or entry as having a p-value<0.10.

## Results

### Nef protein expression by astrocytes is sustained in-vitro and in-vivo

**Fig 1:**
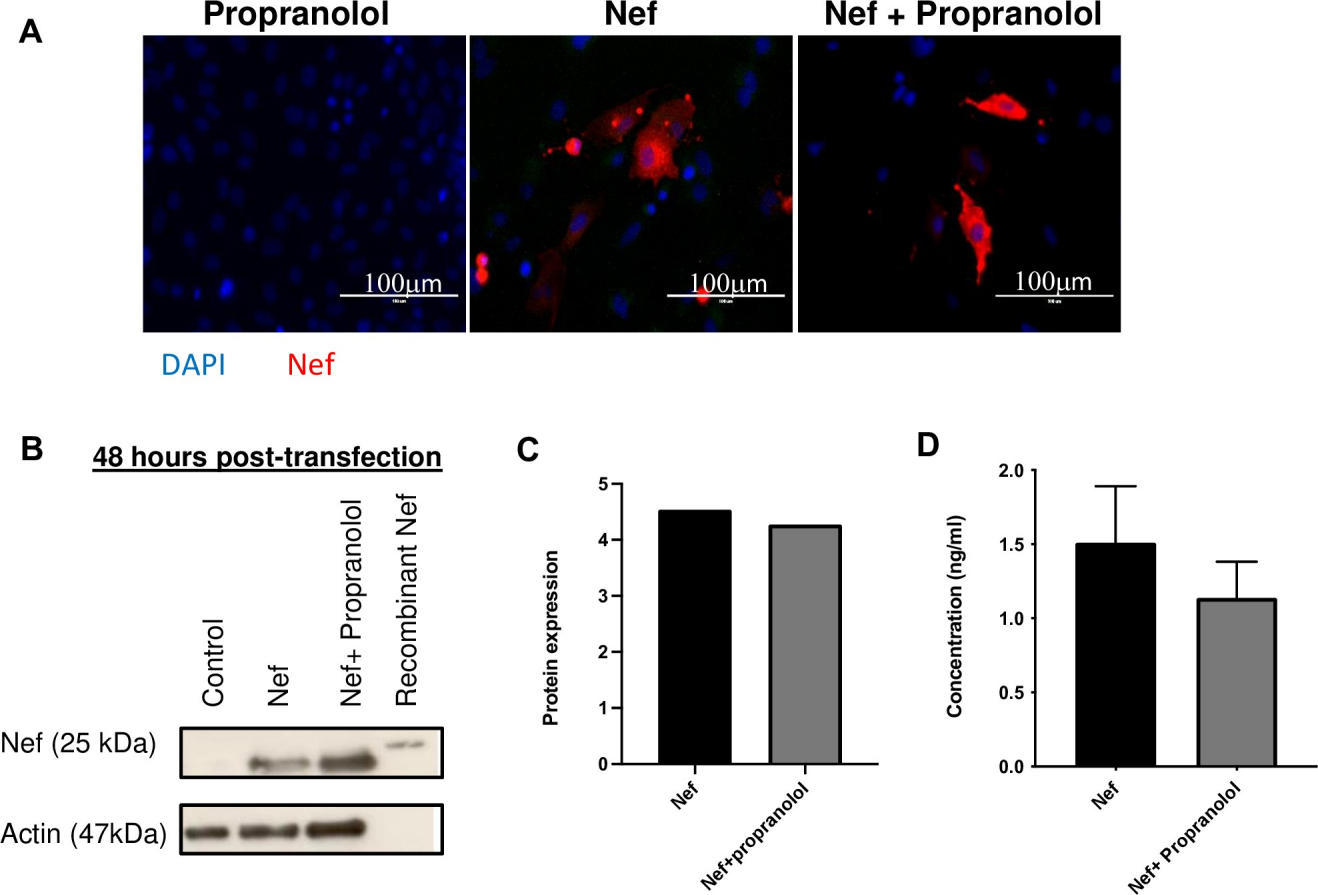
Expression of Nef is sustained for 48 hours even in the presence of propranolol. **(A)** Images are representative of three independent experiments. After transfection with Nef, immunofluorescence of Nef protein (shown in red) was performed on primary astrocytes. Scale bar indicates 100 μm. **(B)** Western blot analysis of Nef 48 hours post transfection showed expression of Nef. **(C)** Densitometry of Western blot shows Nef expression was not reduced by propranolol. Protein levels were normalized to B-actin. **(D)** Nef present in serum was detected by ELISA. Nef levels were normalized to naïve serum concentration and showed no significant difference between groups; propranolol did not significantly change in-vivo Nef expression.

To determine the expression of Nef for the 48-hour duration of this model, we performed immunostaining for Nef on primary rat astrocytes. Fig 1(A) shows representative photos of Nef expression at 48 hours post-transfection; Nef is strongly detected for the Nef + propranolol and Nef groups, but expectedly absent from propranolol only, indicating specificity. To further confirm the specificity of expression, we performed Western blot, which demonstrated Nef expression with a molecular weight of approximately 25 kDa only in the transfected cells as expected, Fig 1(B). The positive control, pure recombinant Nef protein in lane 4 runs moderately higher at 27kDa due to minor sequence variation and the presence of an N-terminal histidine tag used in purification. Densitometric analysis using actin as a reference protein demonstrated that propranolol treatment does not change Nef expression Fig 1(C). To verify the in-vivo expression, we performed ELISA of Nef in serum. ELISA was selected based on our earlier reports that transfected astrocytes secrete Nef [27], and that such astrocytic Nef expression compromises the BBB according to Evans blue dye extravasation and reduction of the tight junction protein claudin-5 [10]. ELISA shows only background expression (~0.2ng/ml) in naïve animals (data not shown), while Nef-treated animals, independent of propranolol administration, showed no significant difference in serum Nef, Fig 1(D). Levels around 1–1.5ng/ml are about 6-10-fold lower than levels reported in HIV-infected, virally suppressed plasma using the same assay [23], consistent with the limited source of Nef-producing cells in our model as compared to an infected human. In brain tissue, we confirmed similar staining for Nef by immunohistochemistry in the hippocampus independent of propranolol treatment (S1 Fig) Together, these results indicate that astrocytes express Nef throughout the experimental protocol, consistent with our prior report that this model maintains Nef expression for seven days in-vitro and in-vivo [27]. Further, treatment with propranolol does not alter the expression, suggesting no effect on the tissue graft or production of Nef by the infused astrocytes.

### Nef plus propranolol treatment decreased IL-1β levels in serum of rats

**Fig 2:**
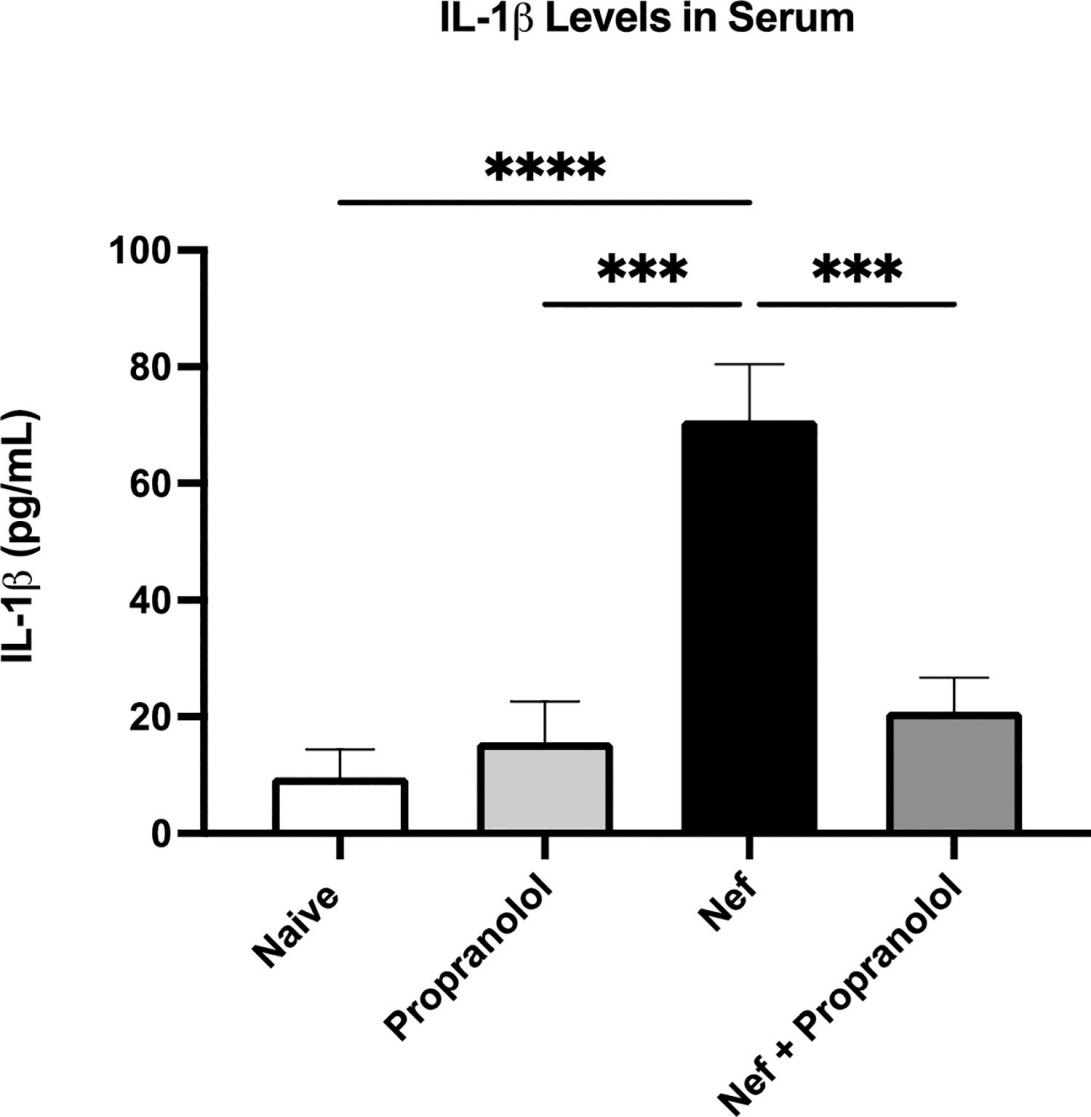
Serum IL-1β levels are decreased in Nef positive rats treated with propranolol. IL-1β measurements in serum via ELISA. Significant upregulation of IL-1β was observed in serum of rats treated with Nef compared to Naïve, Propranolol, and Nef + Propranolol groups. All p-values were two-tailed and statistical significance was defined as *** p-value <0.001 and **** p-value <0.0001.

Using this model, we previously demonstrated Nef expression by astrocytes in the hippocampus caused brain inflammation and spatial learning impairment [27]. A follow up study showed reduced claudin-5 in brain and lung, leaky BBB and lung tissue barriers (Evans blue dye extravasation in Nef-treated rats), and a rise in serum IL-1β expression [10]. These earlier studies showed that Nef expression by hippocampal astrocytes led to macrophage infiltration in brain [27] and peripheral lung and ileal tissues [10]. The infiltration of macrophages, which express β-ARs, in the inflamed areas of the brain, lung, and GI, led us to speculate that signaling by the adrenergic system modulates this response. Therefore, we infused astrocytes with Nef in the right hemisphere of the hippocampus of rats that were pretreated for 24 hours with injection of propranolol (10mg/kg) or saline. A pretreatment model was implemented to test the requirement for β-adrenergic signaling to induce the tissue inflammatory responses we previously reported [10]. Over the next 48 hours, rats received daily injections of saline or propranolol according to treatment group, prior to sacrifice. Upon sacrifice, blood was drawn from the left ventricle to collect serum for the measurement of IL-1β via ELISA. Fig 2 shows the serum measurement of IL-1β in which the Nef group displays strongly upregulated levels of IL-1β. The propranolol and Nef + propranolol groups show no significant upregulation compared to naïve. The results show co-treatment with propranolol blocks the upregulation of IL-1β by Nef.

### Propranolol co-administration blocks the loss of claudin-5 staining caused by Nef

**Fig 3:**
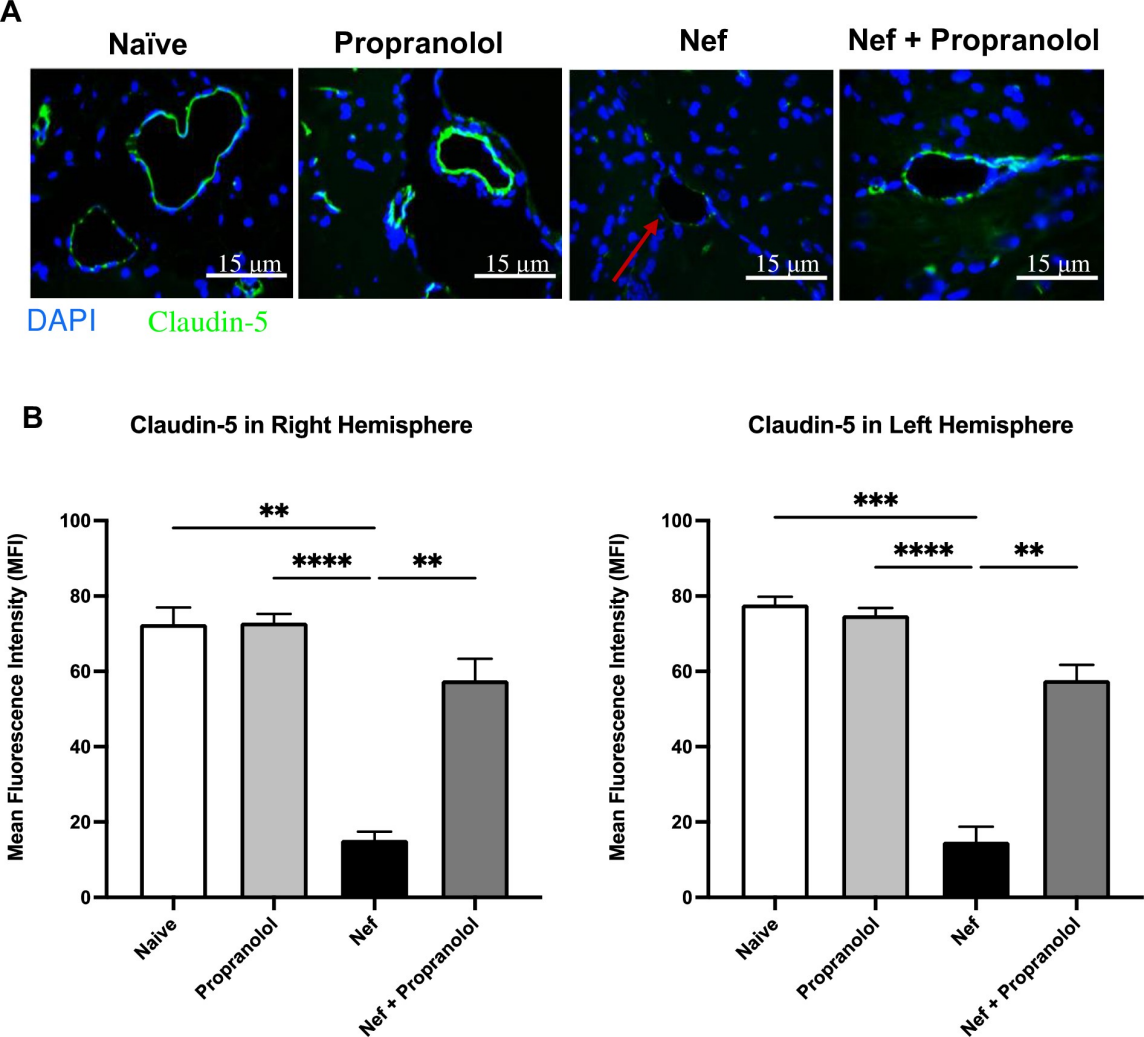
Propranolol attenuates Nef-induced reduction of claudin-5. (A) Representative immunofluorescence images of tight junction protein claudin-5 (shown in green). Absence of strong claudin-5 immunostaining of brain microvessels (red arrow) is predominant in the Nef treated rats. (B) Quantitative analysis showed significant loss of claudin-5 in the Nef-only group. Nef + propranolol treatment partially restored claudin-5 in both hemispheres. Scale bar indicates 15 μm. All p-values were two-tailed and statistical significance was defined as ** p-value <0.01, *** p-value <0.001 and **** p-value <0.0001.

Previously, we were able to demonstrate reduced claudin-5 staining of endothelial barriers in the brain with a loss of BBB integrity [10]. Here, we again measured the claudin-5 (shown in green) as a tight junction protein important to BBB integrity by immunostaining Fig 3(A). Nef expression by astrocytes caused a ~4-fold decrease in fluorescent staining, indicating a loss of claudin-5. However, the rats treated with Nef + propranolol demonstrated a staining intensity and pattern comparable to the naïve animals, consistent with a healthy-appearing and intact tight junction protein staining. Fig 3(B) presents a quantitative analysis conducted in both cerebral hemispheres of the Nef-exposed brain displaying a ~3.5-fold reduced mean fluorescent intensity (MFI) compared to the Nef + propranolol group. These results show that propranolol co-administration prevents Nef-mediated disruption of claudin-5 comparable to normal levels (in the naïve group) in both hemispheres of the brain.

### Histological and immunofluorescence analysis of small intestine show Nef-associated disruption is reduced by propranolol

**Fig 4:**
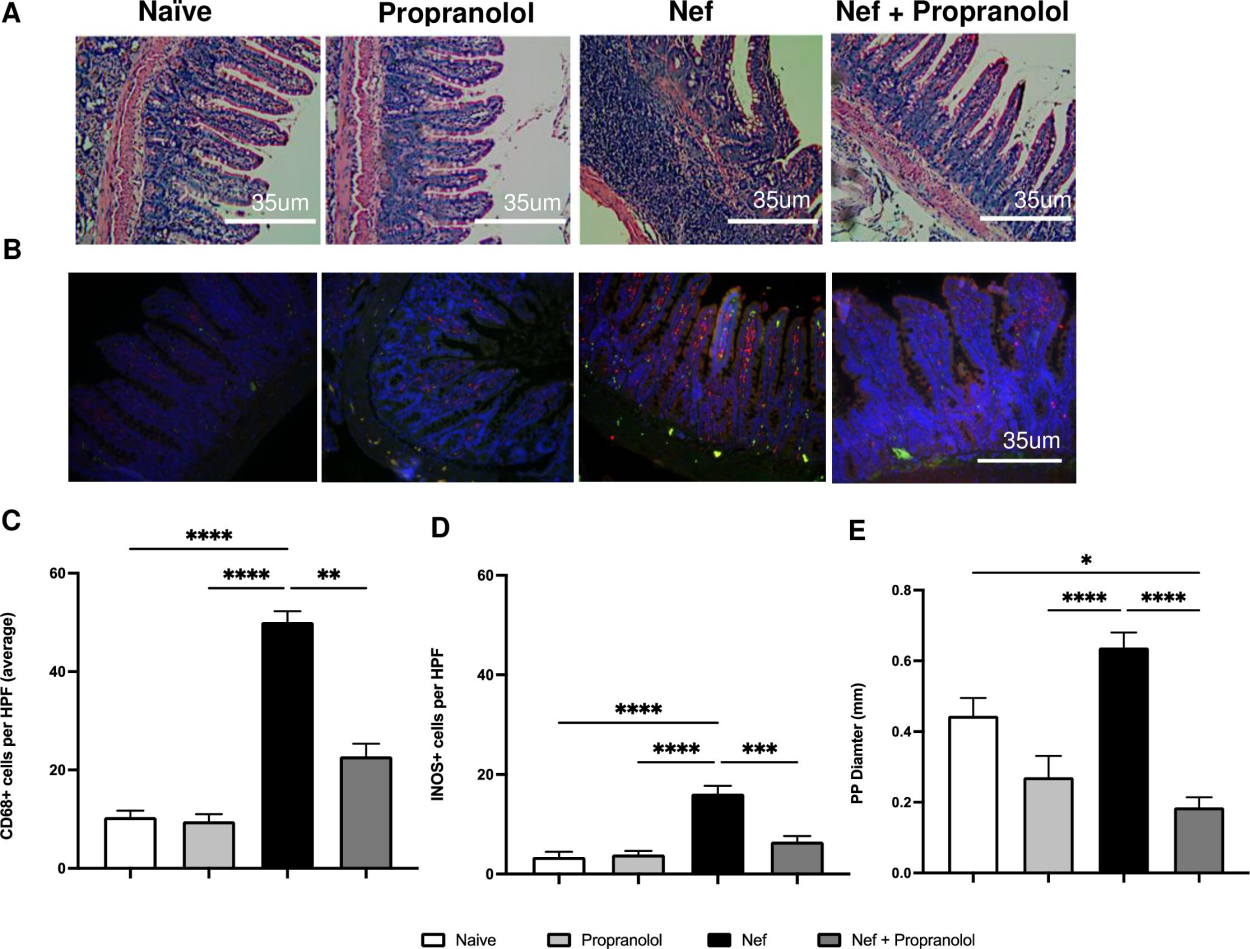
Small intestine inflammation is prominent with Nef exposure and is reduced by propranolol coadministration. (A) Representative photos of Hematoxylin & Eosin staining of the small intestine at (40x). The staining clearly demonstrates the Peyer’s Patches (PP) in the small intestine in the Nef-only group. (B) Immunofluorescence of CD68+ cells (shown in red) and iNOS (shown in green) in the small intestine, blue is DAPI. (C, D) Quantitative results, number of cells counted per high power field (HPF), from the double immunofluorescence evidenced upregulation of CD68+ cells and iNOS in the Nef-only group while CD68+ cells and iNOS remained at normal levels in the Nef + propranolol group. (E) Measurement of PP in the small intestine shows that Nef causes enlargement; the effect is abrogated by propranolol. Scale bar indicates 35um. All p-values were two-tailed and statistical significances were defined as * p-value <0.05, ** p-value <0.01, ***p-values <0.001, and **** p-values <0.0001.

Previously using this model, we found significant inflammation in ileal tissue of the small intestine caused by Nef, including infiltration of macrophages, loss of tissue architecture, and augmentation of Peyer’s Patches [10]. As a follow up, we proceeded to analyze the GI tissue and test if propranolol treatment could abrogate the effects of Nef. Similar to prior findings [10], Fig 4(A) shows disruption of the standard architecture of the small intestine (SI) after Nef treatment compared to the naïve and propranolol-only groups. The Nef-only group shows atrophy and disrupted morphology of the ileal villi, disruption of the muscle layer, and increased infiltration of immune cells. Fig 4(B) depicts merged immunostaining in the SI with the macrophage marker, CD68 (red) and iNOS (green) (see S2 Fig for individual color images). Quantification of CD68 (Fig 4C) shows a 5-fold increase in the group expressing Nef in the hippocampus, while systemic administration of propranolol significantly reduces that increase by 50%.

Similarly, Fig 4(D) shows a significant 8-fold increase of iNOS expression by Nef. Propranolol alone or co-administered with Nef results in no significant increase over baseline (naïve). We analyzed Peyer’s Patches (PP) as an additional parameter in the SI. Peyer’s Patches are lymphoid tissue found in the small intestine. Fig 4(E) shows that the Nef-only group demonstrated significant inflammation shown by an average PP diameter measuring of 0.6mm. This inflammation was greatly reduced it by propranolol coadminstration—mean diameter of PP in the Nef + propranolol group was 0.2mm, significantly less.

### Nef treated rats present thickening of the alveolar space

**Fig 5:**
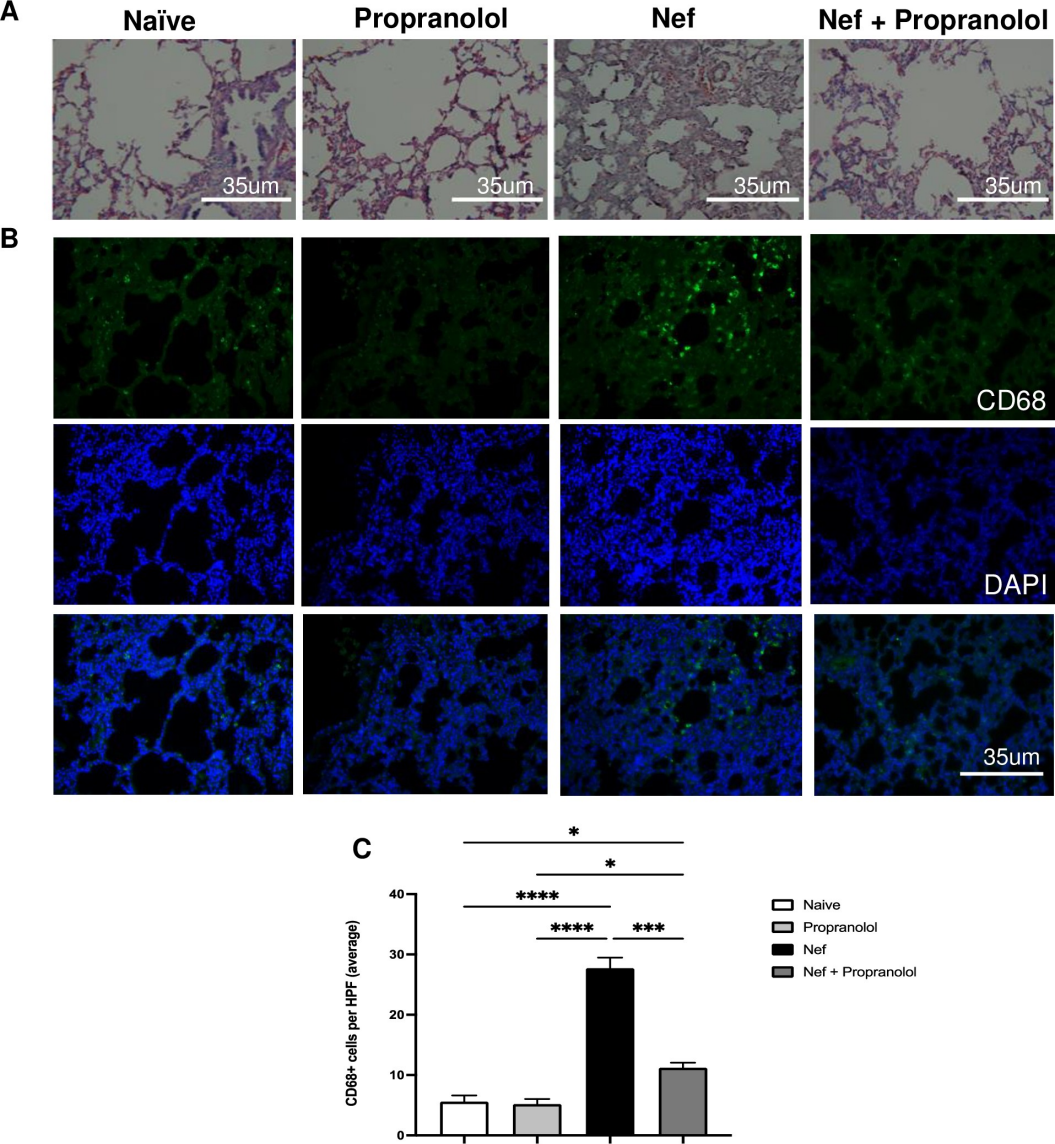
Lung tissue and macrophage infiltration caused by Nef is alleviated through co-administration of propranolol. (A) Hematoxylin & Eosin staining of the lung demonstrated disruption by Nef but healthy tissue in other groups. (B) Representative images of immunofluorescence of CD68+cells (shown in green). (C) Quantification of CD68+ cells confirms that treatment with Nef increases CD68+ cells in the lung, an effect that is neutralized by propranolol treatment. Scale bar indicates 35um. All p-values were two-tailed and statistical significances were defined as * p-value <0.05, *** p-value<0.001, and **** p-value<0.0001.

Lung tissue was also stained with H&E to assess the changes in morphology caused by Nef and to assess if propranolol can reduce the inflammatory response, we previously reported [10]. Fig 5(A) demonstrates the thickening tissue that reduces the alveolar space in the Nef-only group. Analysis of naïve, propranolol only, and Nef + propranolol displays a healthy lung morphology and shows that propranolol treatment reduces the effect of Nef on lung tissue. In addition, to determine if macrophages were part of the immune response present in the Nef-treated group, we conducted immunostaining with CD68+ macrophage marker. Fig 5(B) shows representative photos of the lung tissue with the nucleus stained with DAPI (blue) and CD68+ stained in green. Quantitative analysis proved that macrophages are part of the immune response in the lungs stimulated by Nef, while co-administration of propranolol was able to reduce the effect significantly (Fig 5C). Our results demonstrate that, at the very minimum, activated macrophages are present in the SI and lungs. Furthermore, coadministration of the non-selective beta-blocker propranolol with Nef shows diminished immune activation. This data suggests that Nef expressed in the hippocampal astrocytes can cause peripheral organ inflammation that can be reversed by the use of propranolol, suggesting a role of the SNS in the activation of immune response by Nef.

## Discussion

In the present study, we hypothesized that blocking non-selective β-ARs would inhibit tight junction protein disruption and reduce IL-1β and pro-inflammatory cells (CD68+ cells) response caused by expression of Nef by astrocytes. Coinciding treatment with propranolol resulted in reduced IL-1β, restored claudin-5 at the BBB junctions, and normal (non-inflamed) appearance of tissues in the SI and lungs. These findings suggest the HIV-1 Nef protein is a component of the increased central and peripheral inflammation in our model with the adrenergic system as a mediator of the pro-inflammatory response.

Antiretroviral therapy (ART) can prolong the lifespan of people living with HIV (PLWH). Still, diseases that are not an exclusive result of active HIV replication, such as neurocognitive impairment, cardiovascular problems, SI problems, respiratory disease, liver complications, and cancer, are persistent in PLWH [36–38]. Chronic inflammatory responses are an important part of the secondary complications caused by several HIV proteins whose expressions are not restricted by ART. In the HIV research field, dopaminergic interactions have been well studied, as others have demonstrated a correlation between neuroinflammation and dopaminergic input [39,40]. However, adrenergic signaling is not as well-studied although HIV patients often present damage to areas of the brain, such as hippocampus, where adrenergic signaling is found. More importantly, the adrenergic signaling pathway modulates immunity and may contribute to the chronic inflammation that is a common theme to morbidity among cART-treated individuals [41,42]. In the clinical context, it is known that high levels of NE correlate with a poor response to HIV treatment [43].

Nef alone is neurotoxic and induces the activation of immune cells resulting in the release of various interleukins such as IL-6 and IL-8 and causing neuronal death [43]. This combination of factors can exacerbate catecholamine release, leading to a pro-inflammatory response via upregulation of TNF-alpha, IL-6, and IL-8 [44,45]. Additionally, previous studies in our laboratory have demonstrated that IL-1β is stimulated by hippocampal Nef expression and serves as an inducer that leads to multi-organ pathology. In the present study, we demonstrated that propranolol inhibits the persistent IL-1β secretion by Nef. The effect of lower IL-1β levels results in an abrogated inflammatory effect in the SI and lungs, observed as a lower CD68+ and iNOS+ cell count. Furthermore, we failed to observe the pathological tissue changes (blunted villi and Peyer’s Patches) in the small intestine and interstitial pneumonitis in the lungs when propranolol was co-administered with astrocytes expressing Nef.

Our results suggest that propranolol could be regulating an anti-inflammatory immune response by maintaining the antigen-presenting cells. We propose that exposure to Nef in the brain leads to neuroinflammation that subsequently causes the release of NE. Others have demonstrated Nef to cause neuronal and astrocyte death, creating a toxic environment in the brain. Propranolol can inhibit BBB tight junction protein alterations caused by Nef expression by astrocytes in the hippocampus. Given our current findings of reduced claudin-5 disturbances in the beta-blocker blockade setting, further studies should focus on memory impairment and neuronal damage to further elucidate how antagonism of the β-ARs could reduce the inflammatory response caused by Nef.

In conclusion, our results demonstrated that antagonism of the β-ARs results in a reduction of the Nef-mediated inflammatory effects. These findings suggest a possible mechanism by which the Nef protein could be working in the brain to induce local and peripheral inflammatory responses. Our current study has elucidated a novel finding that could serve as a new therapeutic target to mediate the secondary complications often seen in HIV patients.

## Supporting information

S1 Fig Representative images of immunohistochemical Nef staining in hippocampal coronal slices of rat brains in 40x magnification. (TIF)

S2 Fig Composite of iNOS and CD68 images of Fig 4. (TIF)

S1 Table Sample size of rats per treatment for each figure. (DOCX)

S1 File (XLSX)
