# Supplementary figures and images for: Blockade of beta adrenergic receptors protects the blood brain barrier and reduces systemic pathology caused by HIV-1 Nef protein
Source: PLoS One. 2021 Nov 16;16(11):e0259446. doi: 10.1371/journal.pone.0259446 (PMC8594844; doi:10.1371/journal.pone.0259446)

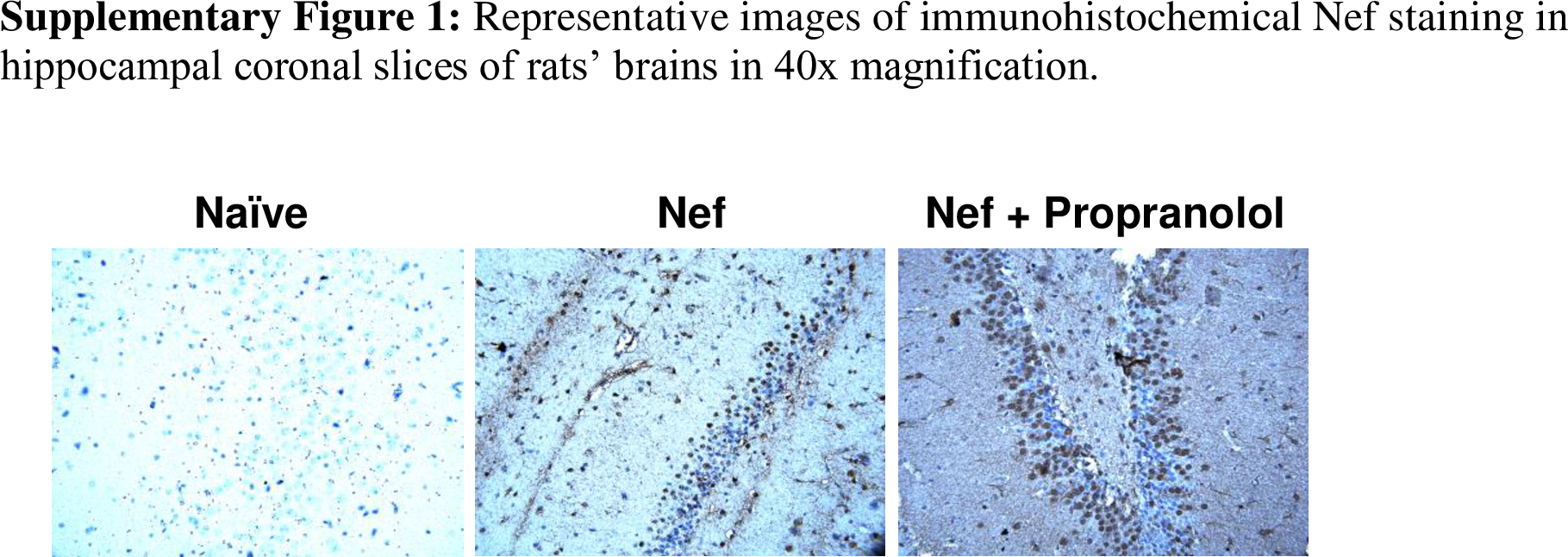

Supplement: S1 Fig — (TIF) [file pone.0259446.s001.tif]

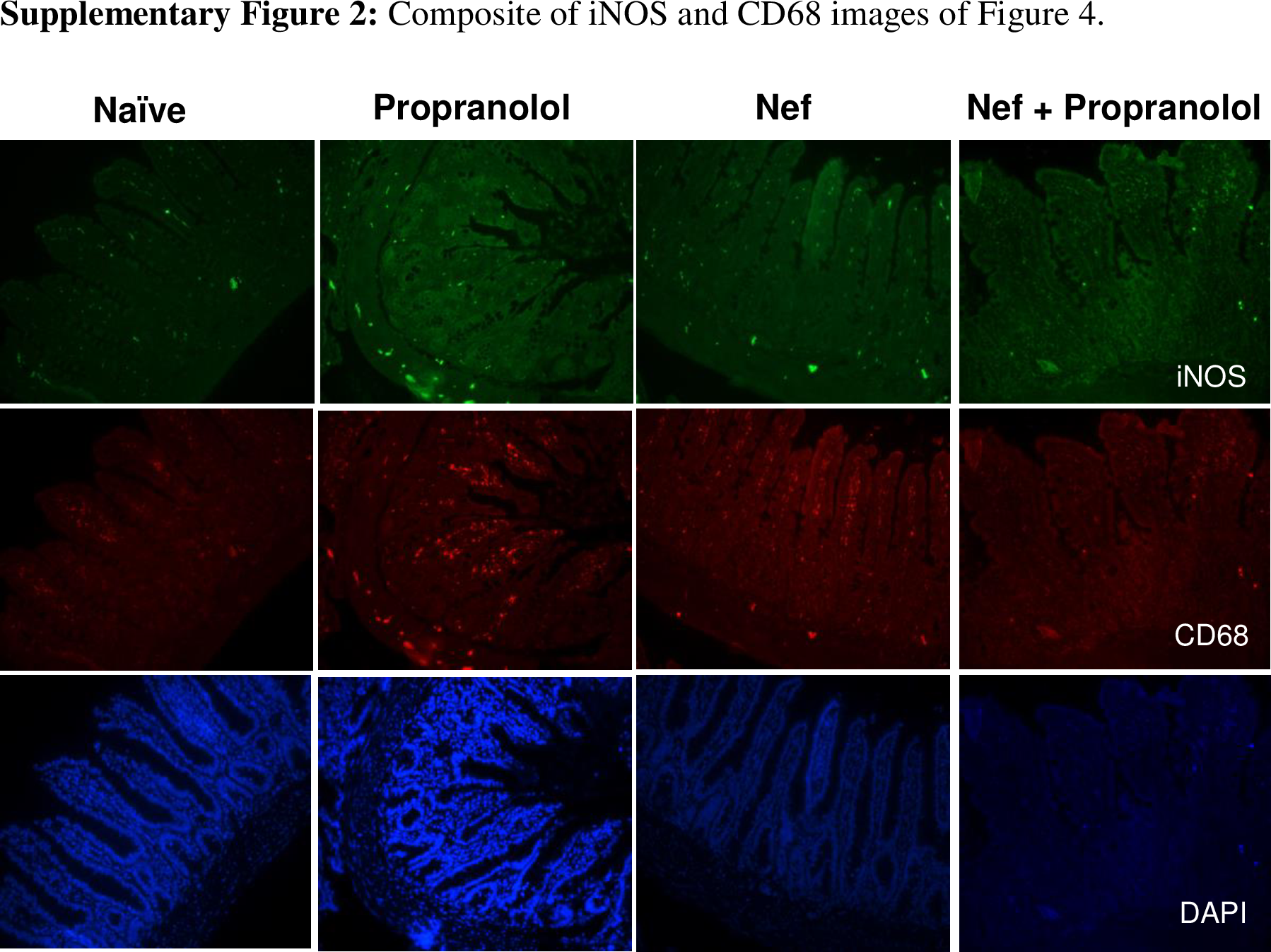

Supplement: S2 Fig — (TIF) [file pone.0259446.s002.tif]
