# Supplementary material for: Blockade of beta adrenergic receptors protects the blood brain barrier and reduces systemic pathology caused by HIV-1 Nef protein
Source: PLoS One. 2021 Nov 16;16(11):e0259446. doi: 10.1371/journal.pone.0259446 (PMC8594844; doi:10.1371/journal.pone.0259446)
Supplement: S1 Table — (DOCX) [file pone.0259446.s003.docx]

**Supplementary Table 1:** Sample size of rats per treatment for each figure.

| **Experiment** | **Number of rats per group** | | | |
| --- | --- | --- | --- | --- |
|  | **Naïve** | **Propranolol** | **Nef** | **Nef + Propranolol** |
| **Figure 1 ELISA** | 7 | N/A | 8 | 9 |
| **Figure 2** | 6 | 4 | 11 | 9 |
| **Figure 3 Right hemisphere** | 4 | 8 | 21 | 9 |
| **Figure 3 Left hemisphere** | 4 | 8 | 14 | 15 |
| **Figure 4 CD68** | 14 | 13 | 16 | 18 |
| **Figure 4 iNOS** | 12 | 13 | 20 | 20 |
| **Figure 4 Peyer’s Patches** | 14 | 19 | 12 | 12 |
| **Figure 5** | 23 | 23 | 25 | 25 |
